# Spinal cord imaging in multiple sclerosis: from diagnosis to disease progression

**DOI:** 10.1007/s00415-026-14122-3

**Published:** 2026-09-30

**Authors:** Tal J. Koren, Jashan Dugal, Dongang Wang, Heidi Beadnall, Chenyu Tim Wang, Yael Barnett, Fernando Calamante, Xavier Montalban, Jiwon Oh, Michael H. Barnett

**Affiliations:** 1 The University of Notre Dame, Sydney, NSW Australia; 2 The Brain and Mind Centre, The University of Sydney, Sydney, NSW Australia; 3 Division of Medicine, University of New South Wales, Sydney, NSW Australia; 4 School of Biomedical Engineering; Brain and Mind Centre, The University of Sydney, Sydney, NSW Australia; 5 Centre d’Esclerosi Multiple de Catalunya, Vall d’Hebron University Hospital, Barcelona, Spain; 6 Division of Neurology, Department of Medicine, St. Michael’s Hospital, The University of Toronto, Toronto, Canada; 7 Neurology Department, The Royal Prince Alfred Hospital, Sydney, NSW Australia; 8 Department of Medical Imaging, St. Vincent’s Hospital Sydney, Sydney, Australia; 9 Sydney Imaging Centre, Sydney, Australia

**Keywords:** Multiple sclerosis, Spinal cord, Magnetic resonance imaging (MRI), Spinal cord atrophy, Expanded disability status scale (EDSS)

## Abstract

Spinal cord (SC) involvement in multiple sclerosis (MS) is common and a major determinant of long-term disability in both relapsing and progressive disease phenotypes. Despite this, SC pathology remains under-assessed in both clinical practice and therapeutic trials, largely due to technical challenges inherent to spinal SC magnetic resonance imaging (MRI) conferred by the cord’s small anatomical size and sensitivity to physiological motion and susceptibility artefact. This review synthesises current understanding of SC involvement in MS, integrating advances in pathophysiology, imaging, and clinical application. We describe the characteristic MRI features of MS SC lesions and their key inflammatory and non-inflammatory differentials, and examine the role of SC imaging in diagnosis, particularly in the context of the 2024 revisions to the McDonald criteria. We highlight the prognostic importance of SC lesions and atrophy, including their association with progression independent of relapse activity, and review the limited and heterogeneous evidence regarding the effects of disease-modifying therapies on SC pathology. We further evaluate current MRI acquisition strategies and consensus guidelines, and discuss emerging quantitative and advanced imaging techniques that detect microstructural and biochemical abnormalities beyond conventional imaging. Together, these developments support a more systematic integration of SC imaging into MS clinical care and trial design.

## Introduction

Multiple sclerosis (MS) is the most common chronic inflammatory disease of the brain and spinal cord (SC), affecting more than 2.9 million people globally[1]. Histopathologically, MS is characterised by multifocal inflammatory demyelination and neurodegeneration that leads to the accumulation of progressive neurological disability in a significant proportion of afflicted individuals [2]. The majority of people with MS (pwMS) present with a relapsing–remitting (RRMS) course. Secondary progression (SPMS), characterised by gradual clinical worsening independent of relapse, ensues in more than half of untreated patients after 15 years[3]. A smaller proportion, approximately 10–15%, have a primary progressive course (PPMS) from onset[1].

SC involvement is a key contributor to disease progression in both RRMS and progressive forms of MS. The presence of SC lesions is associated with increased risk of conversion to MS in patients with radiologically isolated syndromes (RIS) and increased SC lesion burden correlates with worsening disability in pwMS [4, 5]. Indeed, higher Expanded Disability Status Scale (EDSS) scores are heavily weighted towards mobility impairment, which is frequently attributable to SC pathology. Both SC lesions and SC atrophy has long been recognised as an independent prognostic marker for disease progression [6, 7].

Despite this, routine SC imaging, particularly for monitoring of MS disease activity, is not consistently performed in clinical practice, and clinical trials rarely focus on SC lesions or atrophy as imaging endpoints. This gap largely reflects significant technical challenges associated with longitudinal analysis of SC magnetic resonance imaging (MRI) scans. Krieger (2022) recently likened standard clinical SC MRI to “cave paintings”, highlighting the impact of relatively low image resolution (compared with brain MRI), poor signal-to-noise ratio (SNR), and motion artefacts from cardiac and respiratory activity [8]. The assessment of change in SC pathology over time is additionally impacted by the relatively small size of the SC, significant bias introduced by minor inconsistencies in field of view, and inaccurate co-registration. These technical challenges constrain clinical research seeking to correlate SC pathology with clinical progression in MS and evaluate the impact of disease-modifying therapies (DMTs) [9].

In response, there has been a growing focus on improving SC imaging and lesion characterisation in MS, as reflected in the 2024 revisions of the McDonald Criteria [10]. SC lesions in MS have distinctive features, and their identification is crucial for distinguishing MS from other neuroinflammatory disorders, such as neuromyelitis optica spectrum disorder (NMOSD) and myelin oligodendrocyte glycoprotein antibody disease (MOGAD) [11]. Several groups, including the Magnetic Resonance Imaging in Multiple Sclerosis (MAGNIMS) consortium, have developed guidelines to optimise and standardise SC imaging in MS[10, 12, 13], and advanced MRI techniques are increasingly employed in research settings to elucidate the pathophysiology of SC involvement in MS in both SC lesions and normal-appearing white matter (NAWM).

Here, we review the clinical and imaging features of SC disease in MS, with a focus on recent advances. We also discuss the pathophysiology of SC lesions and their evolution in patients with progressive disability, the role of the SC in diagnosis in light of the 2024 revisions of the McDonald Criteria, and radiological differentiation from other inflammatory central nervous system diseases, including NMOSD and MOGAD. We present recommendations for SC imaging sequences in routine MS practice. Finally, we describe advanced MRI sequences and automated analysis techniques that are nearing clinical translation.

## Methods

A PubMed search was conducted in March 2026 to identify articles on spinal cord imaging in multiple sclerosis (MS), with a particular focus on advances in the field over the preceding 4 years. The search used the following keywords: “multiple sclerosis,” “spinal cord,” “imaging,” “MRI,” “lesion,” and “atrophy.” The search identified 382 original English-language articles published between 2022 and 2025, whose abstracts were screened for relevance. Studies were considered eligible if they investigated spinal cord pathology in MS using MRI. Studies were prioritised based on relevance to clinical practice, diagnostic and prognostic significance, longitudinal outcomes, consensus recommendations, and recent advances in quantitative and automated spinal cord imaging. Established knowledge and recent findings were subsequently synthesised and organised into clinically relevant themes as a narrative review.

### Pathophysiology of SC involvement in MS

Discordance between brain and SC involvement in MS is well recognised and likely reflects distinct physiological and immunopathological mechanisms. The grey and white matter interface in the SC is inverted relative to the brain: grey matter surrounds the central canal, while white matter interfaces with the pial-CSF surface [2]. Recapitulating the pathophysiology of brain MS lesions, lymphocytes interact with the SC microcirculation through the venous system, and SC lesions have been shown to exhibit a central vein sign (CVS) on susceptibility-weighted imaging (SWI) [14]. However, subtle distinctions exist. First, the SC has specialised venous drainage, and its lymphatic drainage remains poorly understood[15]. Second, compared with the blood–brain barrier (BBB), the blood–spinal cord barrier (BSCB) shows regional differences in permeability to inflammatory cytokines such as IFNγ and TNFα, as well as the anti-inflammatory IFNα, which may account for the higher prevalence of MS lesions in the cervical spine compared with the remainder of the SC[16, 17].

**Fig. 1:**
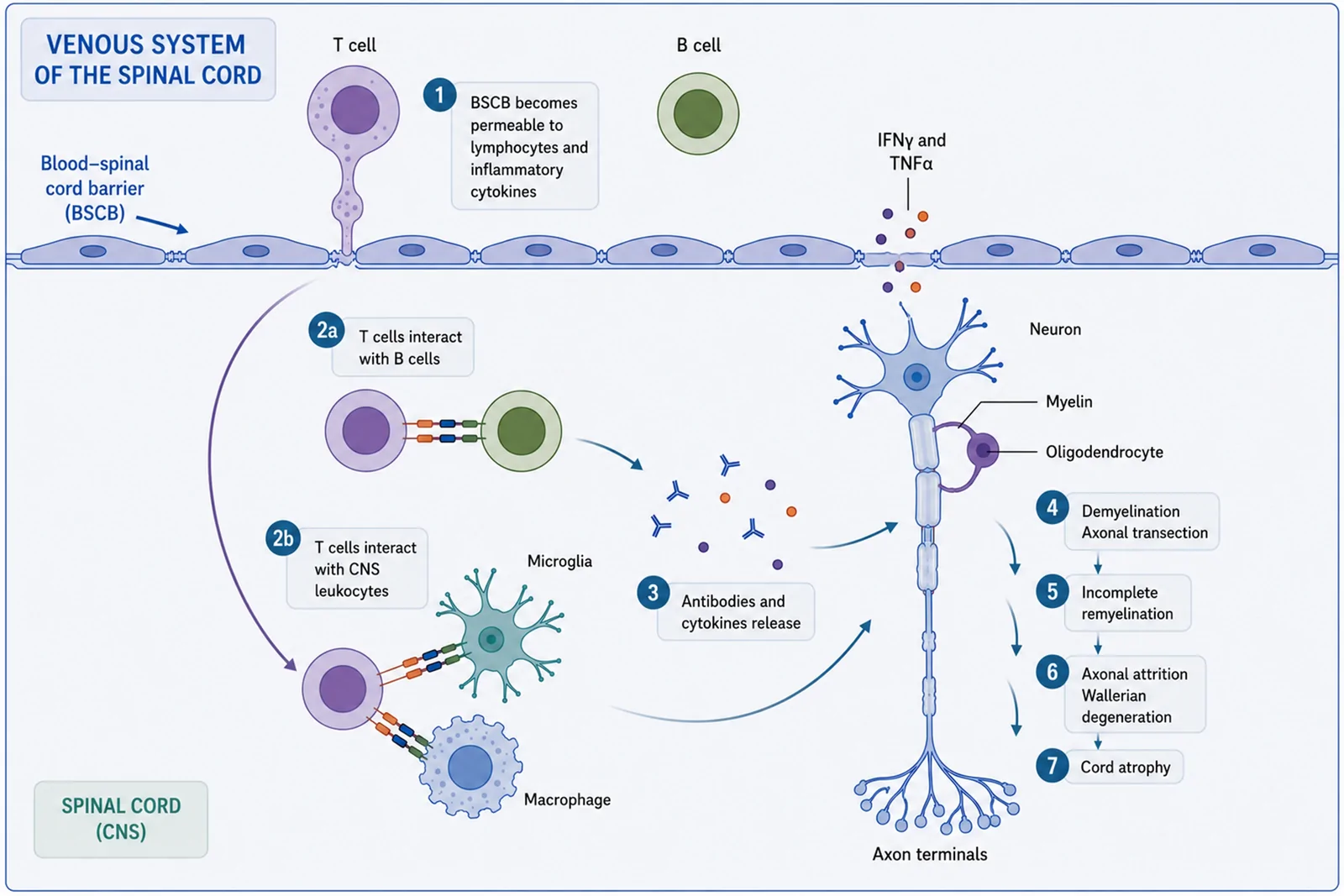
Pathophysiology of spinal cord (SC) inflammation in multiple sclerosis. (1) Auto-reactive T cells, B cells, and monocytes cross a disrupted blood–spinal cord barrier (BSCB) and trigger myelin damage through antibody and cytokine release (2a/2b, 3). (4) Acute lesions show localised macrophage infiltration, demyelination with oligodendrocyte loss, and axonal transection. (5) Chronic lesions are marked by incomplete or absent remyelination and, in some cases, a chronic inflammatory response characterised primarily by microglial activation. (6) Subsequent axonal attrition and Wallerian degeneration lead to progressive SC atrophy. Created in BioRender. Adapted from Höftberger, 2022[26]

The immunopathology of SC lesions largely mirrors characteristic changes observed in brain lesions [2]. Newly forming active lesions are highly cellular and dominated by sheets of macrophages containing myelin debris with sparse T-cell infiltration (Fig. 1). Associated meningeal inflammation is evident in some cases. Demyelinating lesions may involve both white matter and, to a lesser extent, grey matter, particularly in progressive forms of the disease [18]. Acute lesions are associated with significant axonal transection and may be followed by Wallerian degeneration along associated tracts. As in the brain, remyelination of SC lesions is mediated primarily by differentiation of local oligodendrocyte precursor cells, but is more often incomplete[19]. A small subset of SC lesions in proximity to the dorsal/ventral roots or transitional zones may be partially remyelinated by migrating Schwann cells [20, 21]. The molecular composition of Schwann cell-derived myelin is distinct from that of oligodendrocyte membranes, and there may be differences in myelin thickness, internodal length, and possibly conduction efficiency.

The SC pathology that underpins progressive MS is less well defined. Diffuse inflammation may occur, particularly in PPMS, which may not be readily detected by standard clinical MRI sequences[22]. There is a clear lesion-related clinical worsening in many patients with inactive SPMS or PPMS (for example, gradually worsening asymmetric lower limb weakness). Progressive axonal attrition in, around, or along tracts connected to established lesions that may mirror the pathophysiology of chronically demyelinated axons in the brain[23]. While ‘smouldering’ or chronic active lesions (CAL) in the brain are thought to contribute to clinical progression in MS [24], they have only been rarely described in immunopathological studies of the SC [19, 25], and technical constraints, discussed below, limit detection of their likely MRI counterparts (slowly expanding lesions and paramagnetic rim lesions) in vivo. Chronic inflammation in the SC, potentially sustained by neurotoxic cytokines produced by activated microglia and astrocytes, may also lead to diffuse axonal injury and SC atrophy that is independent of lesion burden [2].

### Typical spinal cord lesions in multiple sclerosis

Lesions are present in up to 85% of SC MRI scans in patients with newly diagnosed MS [27, 28]. SC lesions are typically focal, T2 hyperintense, with sharply demarcated borders. They are usually cylindrical or ‘cigar’-shaped on sagittal views and asymmetric ‘wedge’-shaped on axial views. They are often located peripherally, predominantly in the posterior and lateral SC, and are most frequent in the cervical region, particularly at C3–C4 [6]. As many as 76% of patients with RRMS and 90% of patients with PMS have at least one SC lesion on whole-SC imaging [6]. Most lesions measure less than 15 mm in their longest axis and span fewer than two vertebral segments, although individual lesions may coalesce over time. Diffuse SC lesions, areas of abnormal signal intensity without a clearly demarcated border, are more prevalent in people with SPMS (50%) compared with RRMS (18%) [29]. Diffuse lesions are associated with greater disability and may reflect lesion evolution or expansion [28, 29]. However, these lesions are less reliably identified and lack diagnostic specificity. Recent SC disease activity can be indicated by lesional gadolinium enhancement, which is usually nodular but may have a stereotypical incomplete “open ring” pattern[30].

The typical clinical presentation of a new SC lesion is dominated by sensorimotor symptoms and/or signs, which are often bilateral, and may include bowel and bladder dysfunction in patients with thoracolumbar lesions. Localised pain is uncommon, although it may be present in the acute phase. Symptoms are usually subacute, evolving over days and up to 3 weeks [31]. While many SC lesions are symptomatic, in a retrospective study of pwMS, approximately 25% developed asymptomatic SC lesions over a mean period of 17 months [32]. SC MRI activity without clinical symptoms or MRI brain activity has been detected in both RRMS and progressive MS patients, highlighting the importance of SC imaging when assessing pwMS for subclinical disease activity [33, 34].

### Spinal cord lesions in MS diagnosis

The presence of SC lesions can be a critical diagnostic determinant in some pwMS, particularly in patients with non-specific brain white matter lesions. The 2017 McDonald Criteria added the SC as one of four topographies contributing to dissemination of lesions in space [35], extended to five topographies with the inclusion of the optic nerve in the most recent iteration of the criteria [10]. The 2024 criteria also highlight the importance of SC imaging in specific patient subgroups. In patients with suspected PPMS, the presence of two SC lesions is sufficient to demonstrate DIS, without the need for a second topography. Second, in older patients (> 50 years) and in patients with significant vascular risk factors, the presence of additional features, including SC lesions, is recommended to avoid misdiagnosis of microvascular ischaemia [10]. The new criteria also highlight the benefit of MS-specific brain imaging biomarkers for MS diagnosis, such as paramagnetic rim lesions (PRL) and the CVS [36]. However, validation of analogous SC imaging biomarkers is lacking. Future research in this area may have diagnostic implications for patients with atypical features, or those with recurrent transverse myelitis currently diagnosed with double-seronegative NMOSD [37].

### Differential diagnosis

#### Inflammatory myelopathy

**Fig. 2:**
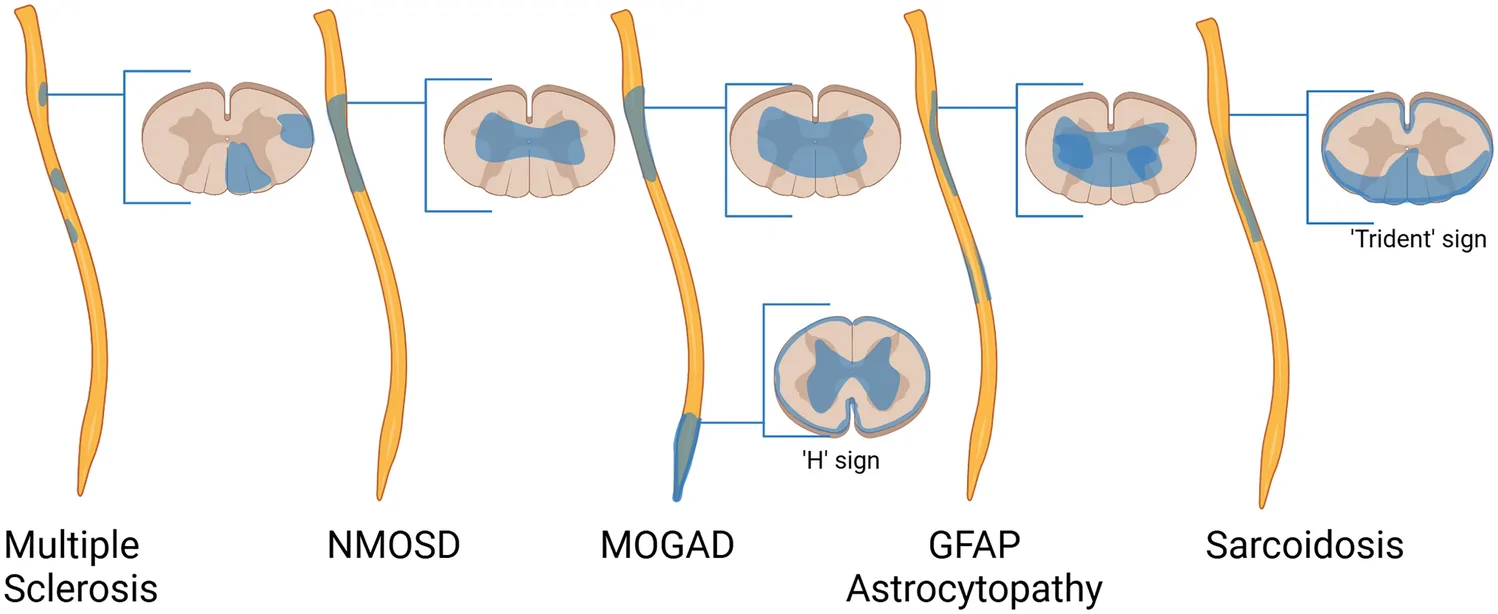
Typical distribution of lesions in inflammatory SC conditions. Legend: NMOSD: Neuromyelitis Optica Spectrum Disorder. MOGAD: Myelin-Oligodendrocyte Glycoprotein Antibody-Associated Disease. GFAP: Glial Fibrillary Acid Protein. Created in BioRender. (2026) https://BioRender.com/g1n1hq8

Accurate characterisation of SC lesions is crucial for distinguishing MS from other inflammatory myelopathies. MS, NMOSD, and MOGAD can all present with focal T2 hyperintense SC lesions (Fig. 2) [31]. Longitudinally extensive SC lesions, which span three or more vertebral segments, should prompt consideration of NMOSD or MOGAD [38]. MS and NMOSD lesions are usually cervical or thoracic, while MOGAD often involves the lumbar SC/conus (Table 1). On axial imaging, NMOSD and MOGAD lesions tend to be central or diffuse, and may be confined to grey matter, whereas MS lesions are more commonly peripheral. MOGAD may also show hyperintensity tracking the anterior and posterior horns, forming the so-called ‘H sign’ [39]. While parenchymal gadolinium enhancement may occur in any of the acute myelitides, complete or incomplete ring enhancement is more typical of MS lesions [40]. In MOGAD, enhancement may be parenchymal and/or leptomeningeal. On follow-up scans, NMOSD lesions may involute but often leave significant atrophy in their wake, while MOGAD lesions may resolve entirely without residual atrophy[41].

**Table 1:** Radiological features of inflammatory myelopathies in the spinal cord

Diagnosis	Multiple sclerosis[31, 40]	NMOSD[31, 38]	MOGAD[39]	Autoimmune GFAP astrocytopathy [43]	Neurosarcoidosis [42]	Paraneoplastic [44]
Lesion length	Short (≤ 2 vertebral segments)	Longitudinally extensive (≥ 3 vertebral segments)	Longitudinally extensive	Longitudinally extensive	Short or longitudinally extensive	Longitudinally extensive
Level	Cervical > thoracic	Cervical or thoracic	Cervical, thoracic or lumbar/conus	Cervical or thoracic	Cervical or thoracic	Cervical or thoracic
Axial distribution	Dorsal or lateral; wedge-shaped	Central	Central	Central	Central or anterior	Lateral, dorsal or central
Enhancement	Parenchymal; acute	Parenchymal; acute	Parenchymal and/or leptomeningeal; acute	Parenchymal and/or leptomeningeal; acute or chronic	Leptomeningeal and/or parenchymal; acute or chronic	Parenchymal; acute
Other radiological clues	Complete or incomplete ring enhancement	Hypointense on T1 imaging, bright spotty lesions	‘H’-shaped grey matter enhancement	Lesion is hazy or ill defined	‘Trident’-shaped enhancement	Lesions can be tract-specific
Follow-up	Lesions may become poorly demarcated; SC atrophy	Lesions decrease in size or fragment; prominent SC atrophy	Lesions often resolve; SC atrophy unusual	Lesions may decrease in size. SC atrophy may be present	Lesions may decrease in size. SC atrophy may be present	Depends on malignancy outcome

*NMOSD* Neuromyelitis Optica Spectrum Disorder, *MOGAD* Myelin-Oligodendrocyte Glycoprotein Antibody-Associated Disease, *GFAP* glial-fibrillary acidic protein, *SC* Spinal Cord

Other inflammatory conditions can also present with SC lesions [40]. Spinal neurosarcoidosis may present with longitudinally extensive transverse myelitis, or rarely tumefactive myelitis, most affecting the cervical or thoracic SC [42]. Leptomeningeal enhancement is common, and axial imaging may show the classical ‘trident sign’, due to central canal and dorsal subpial enhancement, which may persist for months despite immunotherapy [42]. In autoimmune glial-fibrillary acidic protein (GFAP) astrocytopathy, as many as 50% of patients can have abnormal SC imaging, and 30% present with longitudinally extensive transverse myelitis[43]. Lesions are more common in the cervical (71%) and thoracic (65%) SC but can also involve the conus and cauda equina (23%) and usually affect the central grey matter [43]. T2 hyperintensity is usually less focal and hazier. Enhancement may be parenchymal or leptomeningeal and may persist chronically.

Paraneoplastic transverse myelitis has been well described, with anti-neuronal antibodies—including anti-amphiphysin, anti-CRMP-5, anti-Hu, anti-Ri, anti-Yo, or anti-GAD65—present in 80% of proven cases[44]. Lesions are usually longitudinally extensive, predominantly affect the grey matter, may be tract-specific, and often enhance. Transverse myelitis, either isolated or in the setting of acute disseminated encephalomyelitis, may be preceded by a recognised immune stimulus, especially in children, in whom infection or vaccination is identified in up to 75% of cases [45]. Lesions are usually longitudinally extensive and more commonly affect the cervical SC. In adults, myelitis is well described after *mycoplasma pneumoniae* and *COVID-19* infections, amongst other pathogens, although there is often a lack of systematic epidemiological data [46, 47]. *COVID-19* vaccination was temporally associated with more than 500 cases of transverse myelitis in pharmacovigilance datasets [48], though the event is rare, with previously reported rates of 0.28 per million doses [49]. Other vaccinations, including measles and mumps, diphtheria and tetanus, polio and hepatitis B, are only rarely temporally associated with myelitis, although this may be under-reported or under-recognised [50].

#### Non-inflammatory myelopathy

Non-inflammatory myelopathies, such as SC infarcts, spinal arteriovenous fistulae, compressive spondylotic myelopathy, and metabolic, infective, and genetic causes, can also mimic inflammatory SC lesions and should be considered in the differential diagnosis, depending on the clinical context and the presence of clinical and radiological ‘red flags’[31]. A hyperacute (< 12 h) decline in power at onset may suggest an SC infarct, which often presents with severe motor and sensory deficits, autonomic dysfunction, and flaccid tone [51]. Neck or back pain at onset is less common in inflammatory myelopathy but is frequently present in SC infarct and compressive myelopathies. Radiologically, SC infarct is often associated with the classical ‘owl’ eyes or ‘snake’ eyes appearance on axial imaging and an anterior-central ‘pencil’ like pattern T2 hyperintensity on sagittal imaging [52]. Spinal arteriovenous fistulas (AVFs) are arteriovenous shunt lesions that may mimic inflammatory myelopathy, particularly the dorsal intradural subtype (DI-AVF), its most common form. In the typical lesion, the shunt is located in the thoracic or, less commonly, lumbosacral spine near the dural nerve-root sleeve, where a radiculomeningeal feeder artery communicates with a radicular draining vein. DI-AVF usually presents with gradual onset of motor, sensory, and sphincter symptoms and signs that may mimic progressive forms of MS [53]. Clinically, spinal AVF should be considered in patients with a myelopathy who fail to respond to or deteriorate with steroid therapy, as the consequences of a missed diagnosis may be devastating [11, 54]. MRI features include longitudinal T2 hyperintensity within the SC associated with variable intramedullary enhancement, and serpiginous low-signal perimedullary flow voids representing dilated intradural veins. The ‘missing piece’ sign is a relatively specific radiological sign of DI-AVF, characterised by a focal non-enhancing segment(s) within an otherwise enhancing, congested SC, reflecting heterogeneous venous hypertension and impaired venous outflow. Time-resolved magnetic resonance angiography (MRA) and formal digital subtraction angiography (DSA) are usually required to detect the exact location of the fistula and plan treatment [53].

Spondylotic compressive myelopathy may present with insidious onset sensory or motor impairment and pain that localises to the nerve root(s) at the level of compression, long tract signs below the level of injury, and bladder or bowel compromise [55]. MRI may show spindle-shaped central SC T2 hyperintensity with ‘pancake-like’ enhancement just caudal to the site of maximum stenosis that may last for months after surgical decompression [55]. Metabolic insults, such as vitamin B12 and copper deficiencies, may present with tract-specific clinical features such as selective loss of proprioception and vibration due to dorsal column degeneration [56]. These are associated with symmetrical, often longitudinally extensive T2 hyperintensity in the cervico-thoracic SC and, classically, an inverted dorsal T2 hyperintense ‘V’ sign on axial images [57]. Direct infectious myelitis is a well-described but rare complication of infection with multiple pathogens, including *Mycobacterium tuberculosis*, poliovirus, *Brucella*, borreliosis (Lyme disease), HSV1, enterovirus, and the flaviviruses [58]. More recently, pegivirus-associated SC tractopathy has been described in immunocompromised patients[59]. Radiological findings of infectious myelitis vary with aetiology but intramedullary contrast enhancement, cauda equina involvement, and radiculitis may be present[58].

### Spinal cord imaging biomarkers for MS disease progression

The presence of SC lesions confers a worse prognosis across all MS subtypes, from diagnosis to secondary progression. In RIS, the presence of SC lesions is an independent risk factor for conversion to MS at 2 years [5]. Patients with a focal SC clinically isolated syndrome (CIS) are more likely to convert to MS and had a shorter time to conversion, than patients with a non-spinal CIS [60]. SC lesions may also be more strongly associated with disease progression and disability than brain lesions. Multiple studies have shown an association between total SC lesion volume at diagnosis and subsequent disability, as measured by the EDSS score, 9-Hole Peg Test, and 25-Foot Walk Test [4, 6]. The presence of SC lesions, especially in the cervical spine, is also independently associated with conversion to SPMS [61, 62].

There is an increasing emphasis on identifying features of ‘critical’ SC lesions that are strongly associated with greater disability and disease progression. For example, lesions within the cervicomedullary junction may correlate with progressive motor impairment, even with very low or no additional SC lesion burden [63]. Lesion-associated SC atrophy, larger lesion size, and location within the lateral columns on axial imaging are also associated with worse disability [64]. Importantly, both total SC lesion volume and SC lesion number at diagnosis are associated with disability progression independent of relapse activity (PIRA)[65], suggesting that the pathophysiological processes associated with disability progression are incompletely captured by clinically implemented MRI monitoring protocols.

Together with cord lesions, spinal cord atrophy has also been associated with disability progression. PwMS have a smaller cervical SC volume, often measured as cervical SC cross-sectional area (SC-CSA), compared with healthy subjects (73 mm^2^ versus 80.9 mm^2^ across a systematic meta-analysis) [66]. First described by Losseff et al. (1996)[7], SC atrophy in pwMS is accelerated, with a mean atrophy rate of 1.78% per year [66], compared with a ‘physiological’ rate of 0.06% per year in healthy participants [67]. In both RRMS and SPMS, there is an association between SC-CSA and disability, as measured by EDSS and the 25-foot walk test [6, 68]. Reduced SC-CSA also correlates more closely with disability progression than brain atrophy, recapitulating the relatively greater impact of SC versus brain lesion burden on clinical worsening [69, 70].

Accelerated SC atrophy is also a predictor of progression to SPMS. In a retrospective study of more than 360 people with RRMS, significantly faster SC atrophy was observed over 4 years in patients subsequently converting to SPMS (2.19%/year) compared with those who did not (0.88%/year) [71]. Cervical SC atrophy rates are higher in the early (< 3-year duration) inflammatory phase of RRMS (1.81 mm^2^/year) and diminish in later disease (3 − 10 years) (0.93 mm^2^/year), although variable atrophy rates have been reported depending on the measurement methodology [66, 72]. Interestingly, total lesion count and volume do not consistently correlate with the rate of SC-CSA atrophy [6], suggesting that the interaction of focal inflammatory injury, tract-specific vulnerability and diffuse axonal degeneration heterogeneously impact SC integrity.

The specific involvement of SC grey matter has also been investigated. Cross-sectional studies demonstrate reduced cervical and thoracic cord grey matter SC-CSA in MS, with greater involvement in progressive forms of the disease, and stronger associations with disability than either whole-cord or SC WM measures [73, 74]. However, predictive longitudinal studies are lacking and clinical implementation of tract-specific MRI quantitation remains challenging, not least because SC MS lesions impedes reliable grey matter segmentation[75].

### Effects of disease-modifying therapies on spinal cord lesions and atrophy

Despite substantial evidence for the contribution of SC pathology to disability and progression in pwMS, clinical trials have primarily focused on brain imaging outcomes. A 2026 systematic review identified that out of 291 MS clinical trials, only 8 (2.7%) planned to include SC MRI metrics as an outcome[76]. Of these, only three (progressive MS) trials, studying dimethyl fumarate [77], laquinimod [78], and lamotrigine [79], respectively, actually published the results of the SC MRI metrics. None showed a significant difference in cervical CSA atrophy rate or in cervical SC lesion number compared with placebo. In a separate prospective cohort study of pwMS commencing treatment with fingolimod, subjects with multiple SC lesions at baseline were significantly more likely to have EDSS worsening (EDSS change = +0.16, p=0.013) than those without SC lesions (EDSS change = − 0.21, p=0.009), despite suppression of new inflammatory disease activity, suggesting a limited impact of treatment on SC-related PIRA[80]. Conversely, a retrospective study of natalizumab showed that it equally reduced both annualised relapse rate and disability progression (EDSS) in patients with and without SC lesions [81]. In a recent MSBase registry study, the rate of new SC lesion formation did not differ significantly in pwMS treated with high- versus low-efficacy DMTs, (HR=0.99, 95% CI 0.51–1.92, P=0.97), although the study was limited by treatment and imaging heterogeneity [82]. Conversely, a similar single-centre retrospective study found that patients treated with high- and intermediate-efficacy DMTs were significantly less likely to develop new SC lesions at follow-up (HR=0.29, 95%CI 0.12–0.75, p=0.01) compared with a mixed cohort of matched patients treated with low-efficacy or no DMT, but not with low-efficacy DMT alone (HR=1.01, 95% CI=0.19–5.24, p=0.99) [83]. More robust data, including prospective clinical trial outcomes, are required to determine whether DMTs differentially impact SC pathology, given the dominant contribution this makes to motor and sphincter disability in pwMS.

### Spinal cord imaging: MRI approaches in current clinical practice

**Fig. 3:**
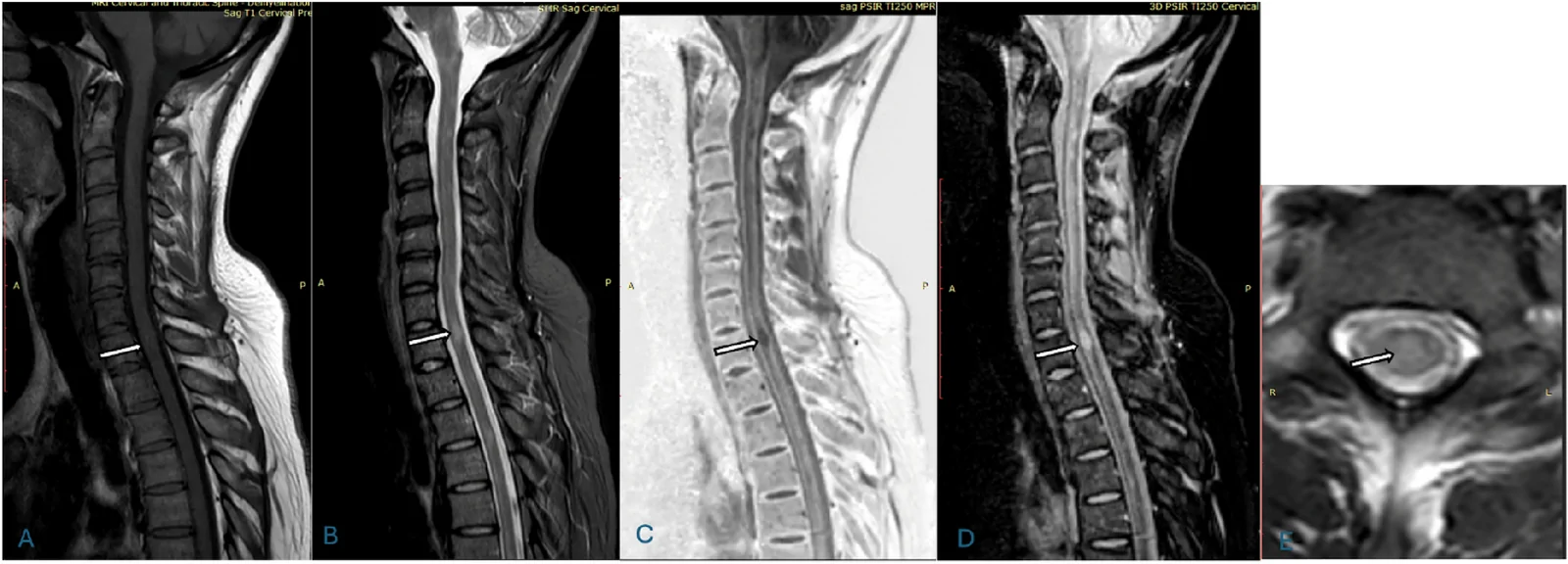
Comparison of appearance of MS lesions on SC MRI sequences. A) 2D T1-weighted sagittal image of the cervical spine in a patient with RRMS. White arrows represent location of known SC lesion. B) 2D T2-weighted short-tau inversion-recovery (STIR) sagittal sequence of the same patient; C) 3D Phase-Sensitive Inversion Recovery (PSIR) sagittal sequence; D) 3D PSIR with black-white inversion. E) T2 axial sequence of the corresponding lesion

Conventional spinal cord MRI predominantly relies on 2D acquisitions, which are relatively robust to motion but remain susceptible to partial-volume effects because of the small dimensions of the cord and comparatively thick slices. T2-weighted and proton density (PD) sequences have traditionally been used to visualise spinal cord MS lesions. PD-weighted imaging may provide greater lesion to cord contrast and is included in many standardised MS imaging protocols. Using a dual-echo turbo or fast spin-echo sequence, PD-weighted images can be obtained from the short echo alongside conventional T2-weighted images from the second echo[84]. More recently, T2-weighted short-tau inversion-recovery (STIR) imaging has been widely adopted, either in place of sagittal T2-weighted imaging or as a complementary sequence, because of its greater sensitivity to MS lesions, especially when diffuse (Fig. 3). However, STIR imaging is prone to flow-related artefacts and false-positive findings [85, 86].

3D acquisitions with near-isotropic voxels can reduce partial-volume averaging and facilitate multiplanar reconstruction and longitudinal co-registration, although they are generally more sensitive to motion and susceptibility artefacts. 3D T1-weighted sequences, including phase-sensitive inversion recovery (PSIR), can visualise MS lesions as high-contrast T1-hypointense abnormalities. 3D-PSIR has demonstrated greater sensitivity than 2D STIR for detecting cervical spinal cord lesions, although results in the thoracolumbar cord have been mixed [87–89]. Similarly, 3D T1-weighted magnetisation-prepared rapid gradient echo (MPRAGE) imaging has shown greater cervical lesion conspicuity and a higher estimated lesion volume burden than T2-FSE and 2D STIR, but it has not yet been widely adopted [90].

Unlike brain MRI, there is no overall advantage of higher field strength (3 T versus 1.5 T) in terms of SC lesion detection in MS [91, 92]. While SNR may be higher at 3 T, greater B0 and B1 inhomogeneity, increased susceptibility to physiological motion, and higher specific absorption rate requirements for SC acquisitions, negate this advantage. Advances in coil technology also diminish the SNR advantages of 3 T imaging [93].

A number of international groups have attempted to create consensus statements and guidelines to standardise SC imaging and lesion identification in MS (Table 2) [10, 12, 13]. The 2024 MAGNIMS-CMSC-NAIMS consensus recommendations on the use of MRI for the diagnosis of multiple sclerosis support using two complementary sagittal sequences, namely gapless T2 FSE/TSE, STIR, PSIR, or MP(2)RAGE images with slice thickness <3 mm, and one axial sequence comprising either T2-weighted or MPRAGE images with slice thickness <5 mm[10]. The whole SC should be imaged, from the cervical SC to the conus medullaris. FLAIR images are not recommended for SC lesion detection. Gadolinium contrast is generally reserved for initial diagnostic scans, suspected acute SC relapse, or suspicion of alternative pathology [10].

**Table 2:** The 2024 MAGNIMS-CMSC-NAIMS recommendations for SC imaging in MS [10]

Field strength	At least 1.5 T
Acquisition	2D or 3D
Slice thickness	Sagittal: <3 mm, no gaps; axial:<5 mm, no gaps
Coverage	Whole SC: cervical SC to conus medullaris
Core sequences	Axial: 2D T2-weighted spin echo or gradient echo Sagittal: Two complementary sequences selected out of T2-weighted, PD, 2D-STIR, 3D-PSIR, 3D-MP(2)RAGE. FLAIR not recommended
Contrast	At diagnosis, in suspected acute relapse, or to exclude alternative pathology
Frequency	At least at diagnosis; Interval frequency not specified

*FLAIR* Fluid attenuated inversion recovery, MP (2) *RAGE* magnetisation-prepared 2 rapid acquisition gradient echoes, *PD* proton density, *PSIR* phase-sensitive inversion recovery, *STIR* short-tau inversion recovery

Double inversion-recovery (DIR) imaging, which suppresses both CSF and normal white matter signal, is used in clinical settings to improve the detection of cortical brain MS lesions. In the SC, a 3D DIR cervical cord sequence demonstrated higher lesion contrast and detected more lesions than conventional 2D T2-weighted imaging [94]. However, DIR imaging is not widely implemented and its use in SC imaging remains investigational.

In CIS and RIS, imaging the entire SC critically assists in establishing the diagnosis of MS and helps to exclude differential diagnoses. Routine imaging of the SC for monitoring has typically been recommended only in specific clinical settings, including spinal-dominant MS, accelerated disability worsening not readily explained by brain MRI changes, and recurrent SC ‘relapses’ or atypical symptoms that may suggest an alternative diagnosis or comorbidity [12]. However, SC lesions may be asymptomatic and remain undetected without routine imaging [95]. There is mixed evidence for the benefit of incorporating SC imaging to routine brain imaging. While some studies suggest that adding SC imaging to routine brain MRI may increase the likelihood of escalating DMT by up to 59% in pwMS [96], others suggest that in the majority of cases, new SC lesions are concomitant with new brain lesions [97]. Given the frequency of asymptomatic SC lesions, we propose that whole-SC imaging every 2–3 years may be a reasonable approach. However, the optimal interval remains uncertain, and this represents expert opinion rather than a consensus recommendation.

### Spinal cord imaging: limitations of current technologies

The above-mentioned MRI approaches are limited by the anatomy and configuration of the SC and its adjacent structures. The SC is surrounded by CSF within the spinal canal, which is itself encased by the thick bones and cartilage of the vertebral bodies. Wide disparities in magnetic susceptibility between the SC, CSF, bone, soft tissue, and air result in significant image distortion and field inhomogeneity [9]. SC imaging is also limited by the SC’s small diameter, which is only 15 mm at its widest point, necessitating the acquisition of thin image slices (1–3 mm) to accurately depict small SC MS lesions. Finally, imaging of the SC is limited by physiological motion. CSF surrounding the SC demonstrates bidirectional, pulsatile cranio-caudal flow driven by the cardiac cycle, contributing to subtle oscillatory motion of the SC within the spinal canal[98]. Furthermore, the cervical and thoracic SC are adjacent to the heart and lung viscera, which independently move in a rhythmic fashion, generating significant movement artefact. While synchronisation with cardiac or respiratory cycles can reduce artefacts, increased scan time limits routine application. Quantitative assessment of SC pathology also presents unique challenges in longitudinal studies. Specifically, SC-CSA is sensitive to neck positioning and slice angulation, necessitating careful quality control and highly consistent acquisition protocols. These technical challenges significantly hamper the ability to accurately identify subtle SC pathology, and are especially problematic for comparative longitudinal studies, where co-registration of image slices is crucial for detecting changes in lesion size or shape.

### Spinal cord imaging: advanced techniques

Quantitative MRI aims to numerically distinguish tissue features and thus increase specificity. Advanced imaging techniques, such as diffusion MRI (dMRI), magnetisation transfer (MT), relaxometry, and MR spectroscopy, are utilised in MS research to better understand SC pathology and disease activity (Table 3)[2, 99]. They are being used to explore why some SC lesions result in disability, while others do not, recognise myelin and axonal injury in structurally normal-appearing white matter, and potentially identify novel radiological biomarkers for future drug development. Nevertheless, the successful implementation of advanced MRI techniques in the SC depends not only on biophysical modelling but also on acquisition and preprocessing strategies that mitigate motion, susceptibility, and distortion. Among the advanced techniques discussed below, prolonged acquisition times, suboptimal scan–rescan reproducibility, and the absence of harmonised multicentre protocols remain major barriers to clinical implementation. The evidence base is further limited by a predominance of small, single-centre, cross-sectional studies and a paucity of longitudinal validation, restricting assessment of biomarker generalisability and clinical utility. Consequently, many promising SC MRI biomarkers remain confined to the research setting rather than serving as clinically deployable outcome measures.

**Table 3:** Quantitative advanced MRI technologies to assess cord pathology in MS

MRI technique	Principle	Typical MS SC findings	Advantages	Limitations
**Relaxometry **[100]	Quantifies intrinsic tissue relaxation properties (T1/T2/T2*); with multi-component modelling, can estimate myelin-associated water fractions	MWF: reduced in cervical SC in PPMS c/w control [102] MVF: reduced in progressive MS, correlates with disability [106]	Provides indirect but biologically relevant proxies of myelin integrity that can be compared across regions and time	Long scan times for multi-component acquisitions Limited protocol standardisation and longitudinal data for the SC
**Diffusion Tensor Imaging (DTI) **[107]	Sensitises the magnetic resonance signal to the random direction of water displacement, providing indirect markers of microstructural tissue organisation	FA: reduced in RRMS and SPMS MD: increased in RRMS and SPMS RD: increased in RRMS and SPMS [108, 109]	Widely available; provides quantitative indices sensitive to microstructural disruption beyond focal T2 lesions	Not pathologically specific. Susceptible to motion and distortion and partial-volume effects
**Multi-compartment diffusion MRI (e.g. NODDI, “ball and stick”) **[113, 114]	Attempts to separate dMRI signal into multiple compartments (e.g., intra-axonal vs extra-axonal vs free water), to improve biological interpretability	NDI: decrease in RRMS[115] ODI: Increased in NAWM in RRMS [114] V_in_: decreased in lesions in RRMS [113] FWW: increased in lesions and NAWM in RRMS [113]	Potentially more sensitive/specific for myelin damage and axonal injury	Requires multi-shell, high angular resolution scans (longer acquisition time, higher motion sensitivity) and more complex post-processing analysis. Limited standardisation across sites and scanners
**Magnetisation transfer (MT)**[116]	Measures the exchange between macromolecule-bound protons and free water to generate myelin-sensitive contrast	MTR: reduced in RRMS and PPMS and in NAWM[118, 119]	Sensitive to myelin damage. Relatively simple to measure and a standard outcome in many MS clinical trials	Semi-quantitative, influenced by acquisition factors and multiple tissue pathologies. Limited longitudinal data in the SC
**Quantitative MT (qMT)**[121]	Model-based quantitative analysis of magnetisation exchange between macromolecular and free-water proton pools	PSR reduced in lesions and NAWM in RRMS[122]	Greater biological specificity for myelin damage than MTR. Potentially better inter-scanner comparability	Longer acquisition times, not widely available. Complex post-processing analysis

*dMRI* diffusion MRI, *DTI* diffusion tensor imaging, *FA* fractional anisotropy, *FWW* free water weight, *MD* mean diffusivity, *MT* magnetisation transfer, *MTR* magnetisation transfer ratio, *MVF* myelin volume fraction, *MWF* myelin water fraction, *MWI* myelin water imaging, *NAWM* normal-appearing white matter, *NDI* neurite density index, *NODDI* neurite-orientation dispersion and density imaging, *ODI* orientation dispersion index, *PSR* pool size ratio, *qMT* quantitative magnetisation transfer, *RD* radial diffusivity, *RRMS* relapsing–remitting multiple sclerosis, *SPMS* secondary-progressive multiple sclerosis, *V*_*in*_ intra-axonal volume fraction

#### Relaxometry and myelin water imaging

Relaxometry, such as T₁ and T₂ mapping, is a quantitative MRI technique that measures intrinsic tissue relaxation time constants sensitive to molecular motion and tissue composition, among other factors [100]. While relaxation metrics aim to identify pathological processes in MS, they are biologically non-specific and are influenced by demyelination, inflammation, oedema, and axonal loss. Myelin water imaging (MWI) techniques, such as Myelin Water Fraction (MWF), are relatively myelin-specific T_2_ map-based parameters derived from water trapped within myelin lipid bilayers [101]. In patients participating in a multicentre clinical trial of glatiramer acetate, MWF in the cervical SC correlated with cross-sectionally derived cervical SC volumes at C2–C3, was reduced in people with PPMS compared with matched healthy controls, and fell by more than 10% over 2 years in the disease group [102]. More recently, quantitative T₁ mapping has emerged as a robust myelin-sensitive measure in the brain in postmortem studies [103], but its interpretation also requires caution given the influence of other non-myelin factors; and SC data remain sparse [104].

More complex multi-component relaxometry models have been explored to improve biological specificity. For example, mcDESPOT (multi-component Driven Equilibrium Single Pulse Observation of T_1_ and T_2_), which estimates the fraction of short-T₂ myelin-associated water, may serve as an indirect surrogate of myelin content and correlates well with histopathologically-proven demyelination in the SC[105]. In vivo, SC putative myelin signals derived from mcDESPOT such as myelin volume fraction (MVF) correlated with disability measures of ambulation and manual dexterity in patients with PPMS [106]. Despite their promise, advanced relaxometry techniques in the SC are constrained by acquisition time, motion sensitivity, and scarce in vivo longitudinal data, which restricts their use to specialised research settings [100].

#### Diffusion MRI (dMRI)

dMRI is a quantitative MRI technique that utilises the microscopic, random movement of water molecules in tissue to generate image contrast. dMRI can provide quantitative information about the magnitude of diffusion across fibrous structures, such as myelin sheaths in the SC, to assess their microstructural organisation and identify damage not detected by conventional MRI sequences [9, 99, 107].

*Diffusion tensor imaging (DTI):* The most widely used model to analyse diffusion data is the diffusion tensor model. Diffusion tensor metrics in the SC of patients with both RRMS and SPMS are abnormal relative to healthy controls. Most notably, fractional anisotropy (FA), a measure of the degree of anisotropic water diffusion, is reduced, while radial diffusivity (RD) (diffusion perpendicular to axons) and mean diffusivity (MD) (the directionally averaged magnitude of diffusion) are increased, compared with healthy subjects, reflecting disrupted fibre organisation and altered water compartmentalisation[108, 109]. Importantly, these diffusion abnormalities extend beyond focal T2-visible lesions into normal-appearing SC white matter and correlate with clinical disability, including EDSS, even in patients with relatively low lesion burdens[110]. Longitudinal studies also demonstrate that SC dMRI metrics evolve over time and are sensitive to progressive microstructural damage, including within normal-appearing SC segments [109]. Recently, Witt et al. (2025) utilised diffusion tractography, which uses diffusion-based directional information to map axonal pathways, to assess tract-specific diffusion abnormalities in the cervical SC of pwMS [111]. However, changes in the conventional diffusion parameters may differ between the brain and the SC, and pathological features of MS, such as inflammation, demyelination, axonal loss, and oedema, may exert confounding effects on individual diffusion metrics [112].

*Non-tensor dMRI models:* Multi-compartment models that aim to disentangle diffusion signal sources from structurally heterogeneous tissue have been developed to enhance biological specificity and sensitivity to subtle or early pathology, such as demyelination or axonal loss. To quantitatively discriminate lesional and non-lesional tissue in the cervical SC, Snoussi et al. used a simplified two-compartment ‘ball-and-stick’ model, which combines an isotropic ‘ball’ to represent the free diffusion compartment, and a highly anisotropic ‘stick’ axial diffusivity (AD) compartment to represent the restricted diffusion motion along axons[113]. In this model, free water weight (FWW), which estimates the relative contribution of free water signal to the total diffusion signal, was significantly increased in the NAWM of the cervical SC of patients with RRMS relative to healthy controls, supporting its role as a quantitative marker of diffuse MS-related pathology. More complex models, such as neurite-orientation dispersion and density imaging (NODDI), enable separation of the diffusion signal into intra-neurite, extra-neurite, and isotropic CSF/free-water compartments. NODDI can be used to estimate parameters, such as neurite density index (NDI) and orientation dispersion index (ODI), providing richer microstructural interpretation. By et al. demonstrated a decrease in NODDI-derived intra-axonal volume fraction (V_in_) within SC lesions and a global increase in ODI across cervical SC white matter tracts in RRMS, demonstrating microstructural characterisation not identified by conventional dMRI metrics[114]. These findings were extended by Collorone et al., who studied a larger cohort and showed widespread reductions in SC NDI that correlated with higher disability scores in RRMS, underscoring the clinical relevance of neurite-specific measures [115]. However, complex models such as NODDI have significantly higher SC MRI acquisition demands (multi-shell, high-b-value data with many diffusion directions) that cannot be reliably deployed in large MS cohorts or clinical trial settings.

#### Magnetisation transfer (MT)

MT imaging exploits the exchange of magnetisation between protons bound in large macromolecules, such as myelin, and protons in free water, and has been extensively used in MS research[116]. MT ratio (MTR), defined as the difference in signal intensity in tissue with and without an MT pulse, has been shown to be particularly sensitive to myelin damage and, potentially, repair [117]. Patients with RRMS and PPMS have lower cervical SC MTR compared with healthy subjects, and lower MTR in the SC is associated with higher EDSS [118]. Patients with recently diagnosed RRMS have a lower MTR across the cervical SC compared with healthy subjects, even when pathological areas (containing T2-visible lesions) are excluded, suggesting a possible role as an MRI biomarker of PIRA [119]. However, conventional MT imaging is highly dependent on acquisition parameters and scanner implementation, and suffers from poor test–retest reliability, limiting multi-site imaging harmonisation in both clinical trials and practice [120].

Quantitative magnetisation transfer (qMT) yields indices that are more quantitative and potentially more specific for myelin[121]. For example, the free pool size ratio (PSR) is reduced in both lesions and NAWM in cervical SC in pwMS compared with control [122]. However, long scan times and more complex post-processing analysis have limited its application in the SC to small, single-centre MS cohorts. Across both MTR and qMT studies, longitudinal validation of MT metrics in the SC is limited. In one prospective study by Oh et al., longitudinal subject-specific slopes for MTR and diffusion metrics (FA, MD, RD, AD) at 2- and 5-year mark, correlated with follow-up EDSS after 5 years. [123]. However, longitudinal studies have been sparse, and further work is needed to corroborate the utility of qMRI metrics for monitoring and predicting disease progression over time in individual patients.

#### Metabolic and biochemical imaging of the spinal cord

Proton MR spectroscopy (^1^H-MRS) allows in vivo assessment of relative intracellular metabolite concentrations, such as N-acetyl-aspartate (NAA), choline and myo-inositol, providing insight into neuronal integrity, inflammation, and gliosis in MS [107]. For example, in a ^1^H-MRS 1.5 T study of mixed RRMS and progressive MS patients, reduced NAA/creatinine levels (which may suggest axonal damage) were associated with SC atrophy and clinical progression at 12-month follow-up [124]. In acute SC relapse, cervical ^1^H-MRS showed that reductions in total NAA were associated with both clinical impairment (9-Hole Peg Test) and diffusion imaging evidence of posterior tract disruption (RD), supporting its sensitivity to acute axonal dysfunction[125]. Reduction in NAA was also observed in the cervical SC of patients with PPMS without significant SC atrophy and correlated with disability, highlighting its possible use as a drug-target biomarker in PPMS [126]. However, long acquisition times, lower spatial resolution, and difficulty differentiating between the intracellular and extracellular pools within an area of interest restrict its use in clinical trials.

Chemical Exchange Saturation Transfer (CEST) MRI is an emerging proton-based imaging technique in which contrast is generated by applying frequency-selective saturation to exchangeable protons (e.g., amide, amine, hydroxyl groups) that subsequently transfer their saturated magnetisation to bulk water[127]. A commonly used variant, Amide Proton Transfer (APT), is sensitive to amide proton concentration and exchange rate, the latter being strongly influenced by tissue pH. Recent SC studies applying APT-CEST in RRMS have shown significantly altered APT signals in NAWM in the cervical SC compared with controls, consistent with diffuse biochemical changes that extend beyond focal lesions [128]. More recently, non-proton-based sodium MRI (^23^Na imaging) has also demonstrated that total sodium concentration, a marker of altered ionic homeostasis that may reflect underlying mitochondrial dysfunction, is increased in the cervical SC of patients with RRMS compared with healthy subjects [129]. Increased sodium signal correlated with reduced FA on diffusion MRI, suggesting diffuse alteration in SC microstructural integrity. Despite the promise of APT-CEST and ^23^Na imaging, validation in larger patient cohorts and protocol standardisation are required before they can be used clinically.

#### Susceptibility-weighted imaging (SWI)

SWI has a high sensitivity to paramagnetic compounds such as deoxygenated blood products and iron. Feasibility for detecting MS-specific susceptibility features in the SC has recently been reported by Jensen-Kondering et al., who described central-vein-like morphology, akin to the brain central vein sign (CVS) [36], in approximately 40% of SC lesions [14]. Furthermore, recent 7 T studies have identified SWI changes at the rim of SC lesions analogous to brain PRLs, which are considered an MS-specific biomarker associated with progressive disease [130]. While histopathological validation of such ‘SC PRLs’ is lacking, SC SWI may ultimately improve pathological lesion characterisation and aid differentiation between MS and non-MS myelopathies. However, clinical translation is limited by motion sensitivity, susceptibility artefacts from surrounding osseous structures, and reliance on high-field strength MRI.

#### Ultra-high-field (UHF) imaging

UHF imaging provides greater anatomical resolution and increased SNR [93]. Using higher spatial resolution at 7 T MRI allows better demarcation of SC MS lesions, with a reported 52% increase in lesion detection compared with 3 T[131]. Combining UHF imaging with SWI may identify MS-specific features such as the CVS and PRLs, discussed above [130]. However, at higher field strengths, SC MRI is more susceptible to image distortion due to increased B_0_ and B_1_ inhomogeneity, and to motion-related phase errors [93]. Additionally, 7 T MRI scanners are not routinely accessible outside research centres. A recently described alternative approach, Ultra-High Contrast MRI sequences, amplifies small intrinsic differences in tissue T_1_ (or T_2_/T_2_*) using inversion-recovery or bipolar-filter techniques, enhancing lesion conspicuity without requiring ultra-high field hardware [132].

### Artificial Intelligence (AI) models in MRI spinal cord analysis

AI-based technologies have been widely applied to brain imaging in both research and clinical settings. In MS, convolutional neural networks (CNN) have been used primarily to automate lesion and brain/brain substructure segmentation, providing quantitative cross-sectional and longitudinal metrics that enhance research workflows and supplement qualitative radiology reporting [133–135]. Most commonly, U-Net based CNN architectures are used for image segmentation. These employ an encoder–decoder structure to learn spatial features and delineate abnormal tissue at the pixel or voxel level. During training, MRI images are paired with expert-annotated lesion masks, allowing the network to iteratively optimise its parameters to recognise patterns associated with MS lesions. Performance is then assessed on previously unseen test data, providing an independent measure of lesion detection and segmentation accuracy.[133]. The application of these tools to SC imaging has been limited by the same technical challenges that affect conventional SC MRI acquisitions, and none are currently implemented in commercial, regulatory-approved AI-based analysis products.

The Spinal Cord Toolbox (SCT) is an open-access collaborative analysis package created to assist the development and validation of imaging tools for multiple SC pathologies[136], including MS. Initially, the SCT implemented DeepSeg, a CNN-based algorithm for segmenting SC MS lesions (‘sct_deepseg_lesion’) in clinical research [137]. Although DeepSeg has good lesion specificity, its precision is reduced in scans with varying lesion sizes and locations, necessitating careful quality control and limiting its use as a fully automated quantitative endpoint for clinical trials. Naga Karthik et al. developed an acquisition**-**specific, U-Net model (‘lesion_ms_axial_t2’) in SCT that offers more robust lesion segmentation across the entire SC on axial T2 data [138]. More recently, SCT has implemented a contrast-agnostic 3D U-Net-based deep learning model (‘lesion_ms’), trained on multi-site, multi-contrast, multi-sequence clinical data, although external clinical validation data are lacking. To achieve longitudinal lesion tracking, these AI models require both cord and lesion co-registration, which is hampered by inter-scan variability due to cord angle and positioning within the spinal canal, and by lesion evolution over time. To date, there are no models that facilitate native longitudinal lesion tracking.

Besides SCT, Tsagkas et al. (2023) developed a fully automated cervical SC compartment segmentation framework (SCORE) using specialised neural network layers (multidimensional gated recurrent units) to achieve high accuracy and reproducibility for grey matter, white matter, and lesion segmentation in pwMS [139]. However, reliance on a specialised, non-standard MRI sequence, 2D Averaged Magnetisation Inversion Recovery Acquisitions (AMIRA), for model training (and validation) limits the generalisability and clinical applicability of this approach. Similarly, Lodé et al. (2025) evaluated a U-net CNN tool trained on combined sagittal T2 and STIR acquisitions to aid clinicians in detecting SC MS lesions, demonstrating improved sensitivity without compromising precision [140]. However, the tool was assessed only as a lesion detection aid and not validated for quantifying lesion volume or for tracking longitudinal lesion activity.

While these developments in AI-based SC imaging analysis are promising, significant limitations, such as poor generalisability, scan–rescan variability, lack of longitudinal lesion tracking, and a paucity of clinical validation hinder their implementation as software-based medical devices in clinical settings. Beyond post-processing and lesion segmentation, AI is increasingly utilised in earlier stages of the imaging pipeline, including acquisition optimisation, accelerated image reconstruction and motion, and artefact correction [141]. These applications may be particularly relevant to spinal cord MRI, where prolonged acquisition and physiological motion remain major barriers to routine imaging.

## Conclusion

SC involvement in MS is common and more closely associated with disability progression than brain lesion burden. SC lesions may be asymptomatic, are prognostically powerful, and have assumed a more critical role in MS diagnosis, especially in patients with progressive disease. Despite this, MS SC pathology remains under-measured in both clinical trials and routine practice due to technical and practical limitations conferred by its small calibre, physiological motion, and surrounding osseous structures. Improvements in the conventional and advanced MRI technologies, and post-processing techniques including AI-based analysis, are beginning to address this unmet need. Newer 3D sequences such as 3D PSIR or MPRAGE variants, which improve lesion conspicuity, are likely to be incorporated into future consensus protocols; and facilitate the provision of quantitative AI-based SC metrics that complement conventional radiology reporting. Advanced SC imaging techniques, such as dMRI, MT, relaxometry, metabolic imaging, and UHF approaches, can unmask microstructural MS SC pathology and improve pathological specificity in research settings. However, they remain constrained by scan time, sensitivity to motion, poor standardisation, and lack of longitudinal data, and are unlikely to see broad near-term clinical translation. Notably, the application of SWI to the SC may have future diagnostic implications if recent studies on SC CVS can be reproduced and clinically validated.

The strength and generalisability of the inferences drawn from this review are constrained by the predominance of small, single-centre and cross-sectional studies, heterogeneity in MRI acquisition and segmentation methods, and limited longitudinal validation of quantitative SC biomarkers.

For now, all patients should have high-quality MRI of their entire SC, with a minimum recommended acquisition including an axial T2-weighted sequence and two sagittal 2D or 3D sequences (among T2-weighted, PD, 2D-STIR, 3D-PSIR or 3D-MP(2)RAGE) (Table 2)[10]. In the absence of specific consensus recommendations, and because a moderate proportion of SC lesions are asymptomatic, we propose routine whole-SC imaging every 2–3 years to maximise the opportunity to escalate DMT in patients with active disease before irreversible disability ensues. Finally, future clinical trials should consider incorporating SC imaging endpoints, such as lesion burden and SC atrophy, as these may assist with the development of targeted DMTs, especially in progressive disease cohorts.

## Data Availability

Data sharing is not applicable to this article as no datasets were generated or analysed during the current study.

## Abbreviations

*1H**-MRS*: Proton MR spectroscopy
*AI*: Artificial intelligence
*AMIRA*: Averaged Magnetisation Inversion Recovery Acquisitions
*APT*: Amide proton transfer
*BBB*: Blood–brain barrier
*BSCB*: Blood–spinal cord barrier
*CEST*: Chemical exchange saturation transfer
*CIS*: Clinically isolated syndrome
*CNN*: Convolutional neural networks
CSF: Cerebrospinal fluid
CVS: Central vein sign
dMRI: Diffusion MRI
DMT: Disease-modifying therapy
DTI: Diffusion tensor imaging
EDSS: Expanded disability status scale
FA: Fractional anisotropy
FLAIR: Fluid attenuated inversion recovery
FSE: Fast spin echo
FWW: Free water weight
MAGNIMS: Magnetic Resonance Imaging in Multiple Sclerosis
mcDESPOT: Multi-component Driven Equilibrium Single Pulse Observation of T1 and T2
MD: Mean diffusivity
MOGAD: MOG antibody associated disease
MPRAGE: Magnetisation prepared rapid gradient echo
MRI: Magnetic resonance imaging
MS: Multiple sclerosis
MT: Magnetisation transfer
MTR: Magnetisation transfer ratio
MWF: Myelin water fraction
MWI: Myelin water imaging
NAA: N-acetyl-aspartate
NAWM: Normal appearing white matter
NDI: Neurite density index
NMOSD: Neuromyelitis optica spectrum disorder
NODDI: Neurite-orientation dispersion and density imaging
ODI: Orientation dispersion index
PD: Proton density
PIRA: Progression independent of relapse activity
PPMS: Primary progressive MS
PRL: Paramagnetic rim lesions
PSIR: Phase-sensitive inversion recovery
PSR: Pool size ratio
pwMS: People with MS
qMT: Quantitative magnetisation transfer
RD: Radial diffusivity
RIS: Radiologically isolated syndrome
RRMS: Relapsing–remitting multiple sclerosis
SC-CSA: Spinal cord cross-sectional area
SC: Spinal cord
SCORE: Spinal Cord Compartment Segmentation
SCT: Spinal cord toolbox
SNR: Signal-to-noise ratio
SPMS: Secondary progressive multiple sclerosis
STIR: Short-tau inversion recovery
SWI: Susceptibility-weighted imaging
TSE: Turbo spin echo
UHF: Ultra-high field
MVF: Myelin volume fraction
V_in_: Intra-axonal volume fraction
